# Health Tract No. 334

**Published:** 1868-09

**Authors:** 


HEALTH TRACT NO. 334.

### BOXING THE EARS

Is an inexcusable brutality ; many a child has been made deaf for life by it, because the "drum of the ear" is a membrane, as thin as paper, stretches like a curtain just inside the external entrance of the ear, there is nothing but air just behind it and any violent concussion is liable to rend it in two, and the "hearing" is destroyed forever, because the sense of hearing is caused by the vibrations of this drum or "Tympanum."

"Picking the ears" is a most mischievous practice, in attempting to do this with hard substances an unlucky motion has many a time pierced the drum and made it as useless as a pierced india rubber life preserver ; nothing sharper or harder than the end of the little finger, with the nail paired, ought ever to be introduced into the ear, unless by a physician ; persons are often seen endeavoring to remove the "wax" of the ear with the head end of a pin ; this ought never to be done, first, because it not only endangers the rupture of the ear by being pushed too far in, but if not so far, it may grate against the drum, excite inflammation and an ulcer which will finally eat all the parts away, especially of a scrofulous constitution ; second, hard substances have often slipped in, and caused the necessity of painful, dangerous and expensive operations to fish or cut out ; third, the wax is manufactured by nature to guard the entrance from dust, insects and unmodified cold air, and when it has subserved its purpose it becomes dry, scaly, light and in this condition is easily pushed outside, by new formations of wax within. Occasionally wax may harden and may interfere with the hearing ; but when this is the case, it is the part of wisdom to consult a physician and let him decide what is the matter and what the remedy ; if one cannot be had, the only safe plan is to let fall into the ear three or four drops of tepid water, night and morning ; the saliva is better still, for it is softer and more penetrating, but glycerine is far preferable to either ; it is one of the blandest fluids in nature and very rapidly penetrates the hardened wax, cools the parts and restores them to a healthful condition ; if in a week, there is not a decided improvement in the hearing, medical advice ought to be had at once, as next to the eye, the ear is the most delicate organ of the body.

